# Sevoflurane postconditioning improves spatial learning and memory ability involving mitochondrial permeability transition pore in hemorrhagic shock and resuscitation rats

**DOI:** 10.1002/brb3.1501

**Published:** 2019-12-12

**Authors:** Li Zhang, Li Huang, Jingxian Wang, Muchun Zhang, Ye Zhang, Xianwen Hu

**Affiliations:** ^1^ Department of Anesthesiology and Perioperative Medicine The Second Hospital of Anhui Medical University Hefei China; ^2^ Key Laboratory of Anesthesiology and Perioperative Medicine of Anhui Higher Education Institutes Anhui Medical University Hefei China; ^3^ Department of Anesthesiology Lu'an Hospital Affiliated to Anhui Medical University Lu'an China

**Keywords:** apoptosis, hemorrhagic shock and resuscitation, mitochondrial membrane potential, mitochondrial permeability transition pore, sevoflurane postconditioning, spatial learning and memory

## Abstract

**Background:**

Hemorrhagic shock induces the cognitive deficiency. Sevoflurane postconditioning has been documented to provide neuroprotection against ischemic–reperfusion injury by suppressing apoptosis. Mitochondrial permeability transition pore (mPTP) plays an important role in apoptosis, but it is unknown if the protective effect of sevoflurane postconditioning on hemorrhagic shock and resuscitation is associated with the change of mPTP opening. Hence, the aim of the study was to find out the precise mechanism of the sevoflurane postconditioning.

**Methods:**

Sprague Dawley rats were subjected to hemorrhage shock for 60 min and then exposed to 2.4% sevoflurane for 30 min at the instant of reperfusion. Additionally, an opener (atractyloside) or an inhibitor (cyclosporine A) of mPTP was used in the animal model before sevoflurane postconditioning. Rats were randomly assigned into groups of Sham, Shock, Shock+Sevoflurane, Shock+Atractyloside, Shock+Sevoflurane+Atractyloside, Shock+Cyclosporin A, and Shock+Sevoflurane+Cyclosporin A treatment. Rat behavior was assessed for 5 days by Morris water maze 72 hr after surgery, and then hippocampus CA1 region was immunohistochemically stained. Brains were harvested 24 hr after surgery to detect the protein expression levels of Bcl‐2, Bax, and cytochrome C by Western blot, the changes of mPTP opening, and mitochondrial membrane potential (MMP).

**Results:**

We found that sevoflurane postconditioning significantly improved rats' spatial learning and memory ability, down‐regulated the expression of Bax, cytochrome C, and caspase‐3, up‐regulated the expression of Bcl‐2, decreased the mPTP opening, and increased the MMP. The neuroprotective effect of sevoflurane postconditioning was abolished by atractyloside, but cyclosporin A played the similar protective role as sevoflurane postconditioning.

**Conclusion:**

These findings proved that sevoflurane postconditioning improved spatial learning and memory ability in hemorrhage shock and resuscitation rats, the mechanism of which may be related to block mPTP opening, increase MMP, and reduce neuron apoptosis in the hippocampus.

## Graphical abstract

Using the model of hemorrhagic shock and resuscitation, the results of this study showed that (a) postconditioning with sevoflurane can improve spatial learning and memory ability by reducing hippocampal neurons loss; (b) sevoflurane postconditioning can increase the expression of anti‐apoptotic protein (Bcl‐2) and decrease the expression of pro‐apoptotic protein (Bax, Cyt C, caspase‐3); and (c) alterations of mPTP opening in the hippocampal play an important role in the protective effect of sevoflurane postconditioning, which may be related with MMP.

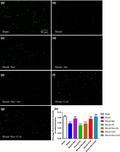

## 1 INTRODUCTION

It is reported that about 5 million people died from trauma each year, and one‐third of them mainly suffered from severe hemorrhagic shock (Murray & Lopez, 1997). Hemorrhage shock leads to inadequate organ perfusion and insufficient tissue oxygenation which cause severe cell hypoxia and apoptosis (Rushing & Britt, 2008). Hippocampus, as a key part of commanding learning and memory, is sensitive to the ischemia and hypoxia (Zhao, Cheng, Ou, Chen, & Ruan, 2012). Therefore, protection of hippocampal neurons against from the damage of hemorrhagic shock is very important.

Sevoflurane is widely used in clinic with many favorable pharmacological features (Delgado‐Herrera, Ostroff, & Rogers, 2001). A number of studies have demonstrated that preconditioning with ischemia or sevoflurane could decrease focal cerebral ischemia and reperfusion injury in rats (Cadet & Krasnova, 2009; Wang et al., 2010). However, the occurrence of ischemia could not predictable in clinical, which limited the applications (Kloner & Rezkalla, 2006). In recent years, some studies have demonstrated that postconditioning with sevoflurane offers neuroprotection following the focal and global cerebral ischemia (Liu et al., 2012; Wang et al., 2010, 2016). But the precise mechanism is unclear.

Mitochondrial permeability transition pore (mPTP) plays an important role in apoptosis. Following ischemia–reperfusion injury, the mPTP will be open, which induces damaging of the neuronal mitochondrial respiratory chain, declining of mitochondrial membrane potential (MMP), releasing of apoptotic factors from the mitochondria and activating of caspase‐3, eventually resulting in apoptosis (Bernardi & Petronilli, 1996; Lim, Davidson, Hausenloy, & Yellon, 2007). Inhibiting mPTP opening by the use of cyclosporin A (CsA; Leger et al., 2011), an mPTP‐specific blocker, has been shown to be neuroprotective in ischemia–reperfusion injury (Ye et al., 2012). Conversely, atractyloside (Atr) was used to open mPTP, which has previously been shown to decrease the protective effect of ischemic preconditioning (Hausenloy, Maddock, Baxter, & Yellon, 2002). Sevoflurane postconditioning may inhibit the excessive release of oxygen free radicals during reperfusion by activating mitochondrial ATP‐dependent potassium ion channels and thus reduce mitochondrial injury (Jiang et al., 2016; Obal et al., 2005). It also has been demonstrated that sevoflurane protects against focal cerebral ischemia and reperfusion injury by involving the opening of mitochondrial membrane permeability transition pore (Morciano et al., 2015; Wang, Wu, Zhou, Yang, & Liu, 2015). However, studies have not examined the role of mPTP opening changes in hemorrhagic shock and resuscitation rats that received sevoflurane postconditioning.

Therefore, the purposes of current study were to investigate if the mechanism of sevoflurane postconditioning improving spatial learning and memory ability of rats following hemorrhagic shock and resuscitation involves the role of mPTP opening changes.

## 2 MATERIALS AND METHODS

### 2.1 Animals

Male Sprague Dawley rats weighing 300–350 g were provided by the Anhui Medical University. All experimental procedures were performed in accordance with the Guide for the Care and Use of Laboratory Animals (NIH publication Number 85‐23, revised 1996) and approved by the Ethics Committee for the use of experimental animals at the second affiliated hospital of Anhui Medical University. This animal experimental study was designed according to the ARRIVE guidelines (McGrath, Drummond, McLachlan, Kilkenny, & Wainwright, 2010).

### 2.2 Establishment of hemorrhagic shock and resuscitation rat model

Rats were housed in a temperature‐controlled (22 ± 1°C, humidity of 40 ± 15%) room with a 12 hr dark/light cycle, fasted for 12 hr before surgery, and free to drink water. All operations were performed under a heating lamp to maintain the rats' rectal temperature at 37.0 ± 0.5°C. The rats were anesthetized by 0.3% sodium pentobarbital (50 mg/kg) via an intraperitoneal injection. An additional dose of pentobarbital 10 mg/kg was administered every 30–45 min to maintain anesthesia (Fang et al., 2006). The trachea was orally intubated, and the breathing was kept spontaneous. The right carotid artery and left jugular vein were separated, and then, the polyethylene catheters were inserted into them for withdrawing and transfusing blood. About 40% of the total blood volume (Total blood volume [ml] = body weight [g] × 6.12%) (Hu, Wang, et al., 2018; Hu et al., 2016) were taken out in 30 min through two‐way automatic infusion pump (Genie Touch) to make the model of hemorrhage shock (Fang et al., 2006). The blood from the right carotid artery was collected in a sterile 20‐ml syringe and perfused through the left jugular vein at 1 hr later. To avoid blood coagulation, all tubes were filled with physiologic saline containing 5 U/ml of heparin and rats were given 500 U/kg of heparin before bleeding (Macias, Kameneva, Tenhunen, Puyana, & Fink, 2004). After reinfusion, all catheters were removed and the rats were observed for 2 hr. The animals were injected with penicillin to avoid infection and returned to their home cages to recovery from anesthesia.

The sham operation is that the right carotid artery and left jugular veins were separated, and then, the polyethylene catheters were inserted into them but not withdrawing and transfusing blood. Rats in sham operation were exposed to mixed gas (95% O_2_ and 5% CO_2_) at the last 30 min of experiment procedures.

### 2.3 Experimental groups

**Figure 1:**
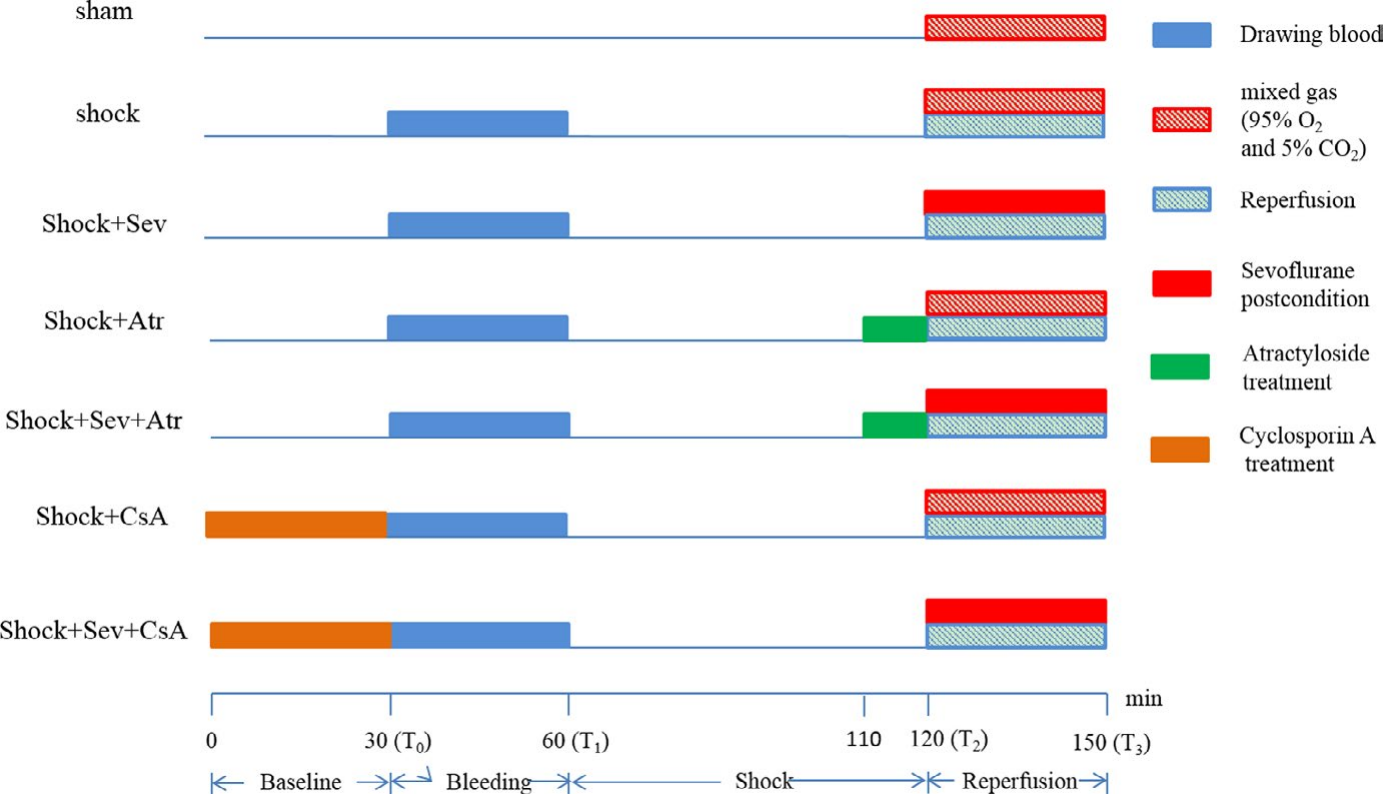
Procedures of hemorrhagic shock and resuscitation rat model and animal grouping. Atr, atractyloside; CsA, cyclosporin A; Sev, sevoflurane postconditioning

Rats were randomly allocated into seven groups (*n* = 18 per group; Figure 1): (a) Sham group: Rats were subjected to procedure described as above without hemorrhage shock; (b) Shock group: Rats were subjected to hemorrhage shock for 60 min and then exposed to mixed gas (95% O_2_ and 5% CO_2_) for 30 min at the same time of reperfusion; (c) Shock+Sevoflurane (Shock+Sev) group: Rats were treated as Shock group but exposed to 2.4% sevoflurane for 30 min at the same time of reperfusion; (d) Shock+Atractyloside (Shock+Atr) group: Rats were treated as Shock group but administered 5 mg/kg Atr (Ye et al., 2012) intravenously 10 min before reperfusion; (e) Shock+Sevoflurane+Atractyloside (Shock+Sev+Atr) group: Rats were treated as Shock+Sev group but 5 mg/kg Atr was intravenously 10 min before reperfusion; (f) Shock+Cyclosporin A (Shock+CsA) group: Rats were treated as Shock group but received 10 mg/kg CsA (Ma et al., 2018; Ye et al., 2012) 30 min before bleeding; (g) Shock+Sevoflurane+Cyclosporin A (Shock+Sev+CsA) group: Rats were treated as Shock+Sev group but 10 mg/kg CsA was administered 30 min before bleeding.

In our study, 126 rats in total were included in the data analysis. Seventy‐two hours after the surgery, six rats of each group were given to Morris water maze test (Hu, Wang, et al., 2018; Hu et al., 2016). After the water maze experiment, rats were deeply anaesthetized and the brain tissues were removed. Twenty‐four hours after the surgery, six brains in each group were used to detect the expression of caspase‐3 by immunohistochemistry. Six hippocampal tissues in each group were used to examine the expression of Bcl‐2, Bax, and Cyt C by Western blot (WB). The mitochondria of the other six hippocampal tissues were extracted to determine mPTP opening and mitochondrial membrane potential (MMP; Lai, Zhang, Su, Cai, & Xu, 2016; Ye et al., 2012).

### 2.4 Postconditioning with sevoflurane, atractyloside, and cyclosporine A administration

Rats were breathing spontaneously during exposure to mixed gas or sevoflurane in the anesthesia plastic chamber. Sevoflurane was delivered by the carrier gases (5% CO_2_ and 95% O_2_, 3.5 L/min total gas flow) through the sevoflurane volatilizer (Vapor 2000) to the chamber (Yue et al., 2008). The concentration of sevoflurane was maintained at 2.4% (Hu, Wang, et al., 2018; Hu et al., 2016; Hu, Zhang, et al., 2018) and measured by an anesthetic monitor (MindrayBeneView T8). The Sham, Shock, Shock+Atr, and Shock+CsA groups were exposed to mixed gas (95% O_2_ and 5% CO_2_), while the sevoflurane treated groups were exposed to sevoflurane at the instant of the reperfusion. The Atr‐treated groups were all administered Atr 5 mg/kg 10 min before reperfusion, and the CsA treated groups were all administered CsA 10 mg/kg 30 min before bleeding (Figure 1).

### 2.5 Hemodynamics and arterial blood gases

During the hemorrhagic shock and resuscitation, the catheter of right carotid artery was connected with pressure transducer to monitoring the mean arterial pressure (MAP, mmHg) which was recorded at a 10‐min interval during operation by Date Record and Analysis system of Power Lab (AD Instruments). The values of arterial blood gas were recorded by withdrawing 0.1 ml blood though the right carotid artery and measured at the beginning of bleeding (baseline, T0), 30 min after bleeding (T1), 60 min after bleeding (T2), 30 min after the reperfusion (T3). The indexes including arterial lactate (Lac, mmol/L), carbon dioxide partial pressure (PCO_2_, mmHg), oxygen partial pressure (PO_2_, mmHg), base excess (BE, mmol/L), and pH were detected by blood gas analyzer (GEM Premier 3500).

### 2.6 Morris water maze test

The water maze apparatus (Zhenghua Biologic) consisted of a 150‐cm‐diameter, 50‐cm‐high circular swimming pool, and a depth of 30 cm covering a black platform 10 cm in diameter. The pool was divided into four quadrants called north, east, south, and west. The platform was placed in a fixed position in the center of the east and submerged approximately 1.5 cm below the surface of the water. Chinese ink was added into water in order to form an obvious contrast with rats to facilitate image acquisition. The spatial acquisition test was started on the 3rd day after reinfusion and carried out for 5 consecutive days. For each trial, the rats were placed randomly into the water facing the wall in each quadrant. Then, the rats were allowed to rest for 60 s. If the rats did not find the platform within 60 s, it was guided to the platform and allowed them to stay there for 15 s to enhance memory. The escape latency (seconds) and swimming speed (cm/s) were observed. Every rat was trained four times per day.

The probe trial was given at 24 hr of the last training to assess memory ability. The platform was removed, and rats were allowed to swim for 60 s in search of it. The swimming distance (cm), the proportion of time spent in the target quadrant (%), and the number of platform crossings (times) were recorded through the computer image analyzer.

### 2.7 Immunohistochemistry

Rats were deeply anesthetized by 3% sodium pentobarbital (60 mg/kg) after the behavioral observation. The brains were removed to keep in 4% paraformaldehyde for 24 hr, dehydrated in graded series of alcohol, embedded in paraffin, and then sectioned into continual 4‐mm thick sections. The sections were incubated in the first antibody the rabbit polyclonal antibody against caspase‐3 (1:300, CST) overnight at 4°C and followed with the second antibody goat anti‐rabbit IgG‐HRP for 30 min at 37°C. The reaction was visualized with 3, 3′‐diaminobenzidine, DAB (DAKO). Data were analyzed by image‐pro plus 6.0 (Media Cybernetics).

### 2.8 Western blot

Twenty‐four hours after reperfusion, the hippocampal tissue was quickly obtained and homogenized using RIPA lysis buffer (Beyotime Institute of Biotechnology). The concentration of total proteins was determined by using BCA method (Thermo Scientific). Samples containing equal amounts of proteins were separated on a 12% SDS‐PAGE gel, transferred to a polyvinylidene difluoride (PVDF) membrane, blocked by nonfat dry milk buffer for 1 hr at 37°C, and then incubated with primary antibody against Bcl‐2 (1:1,000, CST), Bax (1:1,000, CST), cytochrome c (1:1,000, CST), and β‐actin (1:500, Santa Cruz) at 4°C overnight. The membranes were incubated in the second antibody against rabbit or mouse (1:10,000, ZSGB Biologic) for 2 hr at room temperature. Lastly, the immunoblots were visualized with the ECL reagents (Thermo Fisher Scientific) in Tanon full‐automatic imaging system (Bio Tanon). The gray intensities of blots were measured by Image J software.

### 2.9 mPTP opening assay

Mitochondria in hippocampal tissue were obtained by animal cell/tissue active mitochondrial Isolation Kit (Genmed Scientifics Inc.). The mPTP opening was confirmed with calcein‐AM in the presence of cobalt chloride by the purified mitochondrial membrane channel pore assay kit (Genmed Scientifics Inc.) and visualized by fluorescence microscope at an excitation wavelength of 488 nm and emission wavelength of 505 nm. When calcein selectively aggregated inside the mitochondria, it presented green fluorescent staining. When the mPTP opens, it was released from the mitochondria and fluorescent quenching occurred. Changes in mitochondrial fluorescence reflected the degree of mPTP opening. The weaker green fluorescence in mitochondrial indicated the higher degree of mPTP opening. Detailed steps are as follows: Reagent A (10 μl) was added to isolated purified mitochondria (100 μl), mixed, and incubated for 15 min at 37°C in no‐light conditions. Then, the supernatant obtained by centrifugation for 5 min at 16,000 *g* was discarded. Preheated Reagent C (200 μl) was added to the isolated mitochondria, mixed, and centrifuged for 5 min at 16,000 *g*, and obtained supernatant was discarded. Preheated Reagent C 100 μl was added to the mitochondria and mixed. Lastly, the fluorescence intensity of mitochondria was imaged using fluorescence microscope.

### 2.10 MMP detection

Mitochondrial membrane potential (MMP) was measured with a unique cationic dye of 5,5′,6,6′‐tetrachloro1,1′,3,3′‐tetraethyl benzimidazol carbocyanine iodide (JC‐1; Genmed Scientific Inc.) and observed by fluorescence microscope. In living cells, the MMP was high and the mitochondria presented red fluorescence due to JC‐1 accumulated in the mitochondrial matrix to form JC‐1 aggregate, which had the maximum absorption/emission wavelength of 585/590 nm. When the mPTP opening, the MMP was dropped and the red fluorescence was reduced because JC‐1 cannot assemble in the mitochondrial matrix. Thus, the changes of the MMP could be detected by changes of the red fluorescence in mitochondria.

### 2.11 Statistical analysis

All the data were analyzed by two people who were blinded to the specific groups. Unpaired *t* test was used for comparison between two groups. One‐way analysis of variance (ANOVA) followed by a Tukey's test or two‐way ANOVA followed by Bonferroni's test was used for multiple comparisons of more than two groups. Repeated measures of variance were performed to analyze the hemodynamics, arterial blood gases, and the measurements in MWM task at different time points. All analyses were performed with SPSS 10.0, and data were expressed as mean ± *SEM*. *p*‐values < .05 were considered statistically significant.

## 3 RESULTS

### 3.1 Mean arterial pressure and arterial blood gases

**Figure 2:**
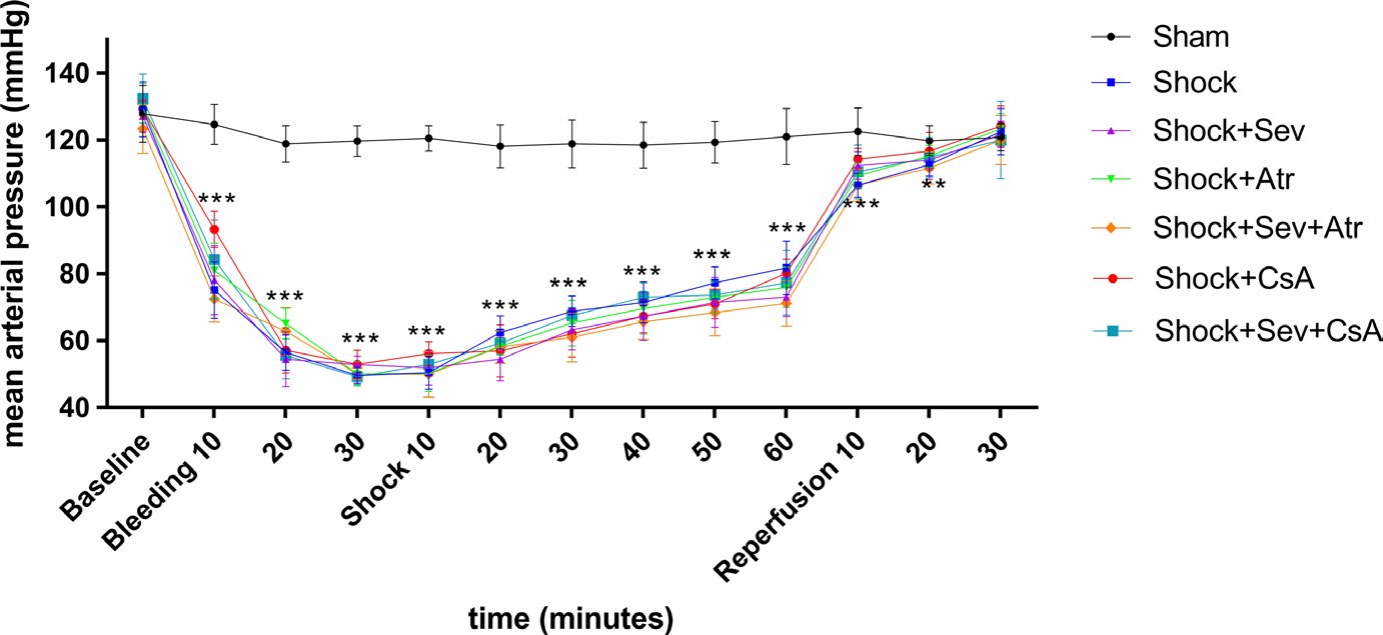
Mean arterial pressure during hemorrhage shock and resuscitation in seven groups. Compared with the baseline period, the mean arterial pressure of six groups was <50 mmHg except for Sham group. Then, the mean arterial pressure returned to baseline level after resuscitation. *N* = 6 in each group, values are presented as mean ± standard error of mean, *p < .01 and **p < .001 versus baseline level

The values of mean arterial pressure (MAP) at the baseline were indistinguishable among all the groups (Figure 2). Compared with the baseline value, MAP value in the Sham group was not significantly changed during the whole experimental period. While compared with the baseline values, the mean arterial pressure (MAP) values of the shock‐induced groups were significantly decreased with or without sevoflurane postconditioning, starting from the first 10 min following bleeding till 10 min before the reinfusion completion (Figure 2; *p* < .01 or *p* < .001), and finally recovered to the base level after 30 min reperfusion. In addition, the severe hypotension was keeping throughout the whole period of shock, but there were no significant differences between shock‐induced groups during the phase of hemorrhage shock.

The values of pH, base excess (BE), lactic acid (Lac), arterial carbon dioxide partial pressure (PCO_2_), and oxygen partial pressure (PO_2_) at the baseline were no different among all the groups. Compared with the baseline values, the four indexes in the Sham group were not significantly changed during the whole experimental period (Table 1). Compared with the baselines, Lac was increased while the pH, BE, and PCO_2_ were reduced at the end of both bleeding and shock (Table 1;* p* < .05, *p* < .01 or *p* < .001). The indexes above were recovered to the basic level after 30 min reperfusion in shock‐induced groups. However, during the whole experimental period, no significant differences in PO_2_ were observed among all groups. Compared with the baselines, PO_2_ at 30 min after reperfusion was significantly increased in all groups (Table 1; *p* < .001) because higher concentration of O_2_ was inhaled during reperfusion.

**Table 1:** The changes of pH, base excess (BE), the lactic acid (Lac), arterial carbon dioxide partial pressure (PCO_2_), and oxygen partial pressure (PO_2_) during and after hemorrhagic shock and resuscitation in seven groups

Index	Group	Baseline	30 min after bleeding	60 min after bleeding	30 min after reperfusion
pH	Sham	7.42 ± 0.02	7.41 ± 0.04	7.39 ± 0.02	7.40 ± 0.04
	Shock	7.41 ± 0.01	7.33 ± 0.02** ^,^ ††	7.34 ± 0.05** ^,^ †	7.41 ± 0.03
	Shock+Sev	7.41 ± 0.03	7.34 ± 0.06* ^,^ †	7.32 ± 0.05** ^,^ †	7.42 ± 0.03
	Shock+Atr	7.42 ± 0.03	7.34 ± 0.04** ^,^ †	7.34 ± 0.03** ^,^ †	7.40 ± 0.04
	Shock+Sev+Atr	7.42 ± 0.02	7.35 ± 0.04** ^,^ †	7.29 ± 0.02** ^,^ ††	7.41 ± 0.04
	Shock+CsA	7.42 ± 0.04	7.36 ± 0.03* ^,^ †	7.30 ± 0.05** ^,^ ††	7.41 ± 0.03
	Shock+Sev+CsA	7.42 ± 0.04	7.30 ± 0.04** ^,^ ††	7.35 ± 0.05* ^,^ †	7.39 ± 0.05
BE (mmol/L)	Sham	1.4 ± 0.6	3.4 ± 0.8	3.2 ± 0.4	3.8 ± 0.9
	Shock	1.3 ± 0.2	−9.2 ± 0.7*** ^,^ †††	−5.8 ± 0.7*** ^,^ †††	1.7 ± 0.4
	Shock+Sev	1.6 ± 0.4	−9.4 ± 0.8*** ^,^ †††	−5.2 ± 0.7*** ^,^ †††	1.7 ± 0.3
	Shock+Atr	1.6 ± 0.7	−9.0 ± 0.5*** ^,^ †††	−4.6 ± 0.4*** ^,^ †††	2.4 ± 0.5
	Shock+Sev+Atr	1.7 ± 0.3	−8.6 ± 0.9*** ^,^ †††	−5.5 ± 0.8*** ^,^ †††	2.0 ± 0.5
	Shock+CsA	1.1 ± 2.0	−9.9 ± 1.1*** ^,^ †††	−5.0 ± 0.9*** ^,^ †††	2.0 ± 0.7
	Shock+Sev+CsA	1.3 ± 2.3	−9.8 ± 1.0*** ^,^ †††	−5.2 ± 1.1*** ^,^ †††	1.4 ± 0.6
Lac (mmol/L)	Sham	1.1 ± 0.2	1.9 ± 0.2	1.9 ± 0.2	1.7 ± 0.4
	Shock	1.5 ± 0.2	10.5 ± 1.0*** ^,^ †††	5.8 ± 0.4*** ^,^ †††	3.1 ± 0.3
	Shock+Sev	1.6 ± 0.3	8.8 ± 0.4*** ^,^ †††	5.6 ± 0.6*** ^,^ †††	2.0 ± 0.3
	Shock+Atr	1.1 ± 0.3	9.5 ± 0.6*** ^,^ †††	6.1 ± 0.5*** ^,^ †††	2.4 ± 0.8
	Shock+Sev+Atr	1.4 ± 0.4	10.3 ± 0.5*** ^,^ †††	6.0 ± 0.4*** ^,^ †††	2.2 ± 0.3
	Shock+CsA	1.2 ± 0.4	10.6 ± 1.1*** ^,^ †††	5.4 ± 1.0*** ^,^ †††	1.6 ± 0.4
	Shock+Sev+CsA	1.2 ± 0.4	10.6 ± 0.8*** ^,^ †††	5.9 ± 0.9*** ^,^ †††	1.8 ± 0.5
PCO_2 _(mmHg)	Sham	46.7 ± 7.2	51.7 ± 6.7	41.0 ± 2.0	43.3 ± 2.5
	Shock	43.0 ± 3.6	24.0 ± 2.0*** ^,^ †††	29.7 ± 2.1*** ^,^ †††	38.7 ± 3.1
	Shock+Sev	42.3 ± 3.1	26.0 ± 1.0*** ^,^ †††	31.7 ± 3.5*** ^,^ ††	41.0 ± 2.0
	Shock+Atr	41.3 ± 2.1	26.3 ± 2.5*** ^,^ †††	29.7 ± 2.1*** ^,^ †††	39.0 ± 2.0
	Shock+Sev+Atr	42.0 ± 3.0	27.0 ± 1.0*** ^,^ †††	31.7 ± 1.5*** ^,^ ††	42.7 ± 3.1
	Shock+CsA	41.0 ± 2.2	22.8 ± 4.1*** ^,^ †††	33.0 ± 4.4** ^,^ ††	38.5 ± 2.7
	Shock+Sev+CsA	42.8 ± 4.5	21.7 ± 2.7*** ^,^ †††	30.5 ± 1.9*** ^,^ †††	38.5 ± 4.7
	Sham	78.4 ± 6.7	80.4 ± 5.2	78.9 ± 3.9	106.0 ± 6.2***
PO_2 _(mmHg)	Shock	80.6 ± 9.1	81.8 ± 6.5	84.1 ± 7.2	107.5 ± 11.0***
	Shock+Sev	77.9 ± 4.1	81.4 ± 4.4	78.0 ± 4.1	105.3 ± 7.01***
	Shock+Atr	81.5 ± 5.0	82.3 ± 3.8	80.4 ± 6.3	101.9 ± 5.3***
	Shock+Sev+Atr	81.6 ± 7.0	79.8 ± 7.3	79.3 ± 3.5	103.6 ± 5.5 ***
	Shock+CsA	79.7 ± 1.0	82.3 ± 4.1	84.8 ± 3.1	102.8 ± 3.1***
	Shock+Sev+CsA	82.5 ± 3.9	83.0 ± 5.4	83.7 ± 6.1	104.8 ± 5.4***

Note

*N* = 6 in each group. Values are presented as mean ± standard error of mean.

* *p* < .05, **p < .01, and ***p < .001 versus baseline level, ^†^ *p* < .05, ^††^ *p* < .01, and ^†††^ *p* < .001 versus Sham group.

Animal rectal temperature was monitored and maintained at 37.0 ± 0.5°C (Table 2). Compared to the sham group, there were no significant differences between other groups during the whole experimental period.

**Table 2:** The changes of the temperature (*T*) during and after hemorrhagic shock and resuscitation in seven groups

Index	Group	Baseline	30 min after bleeding	60 min after bleeding	30 min after reinfusion
*T*	Sham	37.13 ± 0.14	36.80 ± 0.27	36.70 ± 0.15	36.94 ± 0.17
	Shock	37.06 ± 0.23	36.73 ± 0.29	36.50 ± 0.22	36.81 ± 0.19
	Shock+Sev	37.15 ± 0.32	36.64 ± 0.41	36.60 ± 0.23	36.71 ± 0.26
	Shock+Atr	37.24 ± 0.24	36.60 ± 0.23	36.65 ± 0.18	36.75 ± 0.26
	Shock+Sev+Atr	37.25 ± 0.19	36.63 ± 0.17	36.61 ± 0.19	36.74 ± 0.26
	Shock+CsA	37.09 ± 0.18	36.74 ± 0.46	36.57 ± 0.27	36.73 ± 0.31
	Shock+Sev+CsA	37.15 ± 0.52	36.84 ± 0.26	36.76 ± 0.42	36.81 ± 0.38

Note

Temperatures at the beginning of bleeding (baseline, T0), 30 min after bleeding (T1), 60 min after bleeding (T2), and 30 min after the reperfusion (T3) were measured. *N* = 6 in each group. Values are presented as Mean ± *SD*.

### 3.2 Effect of sevoflurane postconditioning on the spatial learning and memory ability

**Figure 3:**
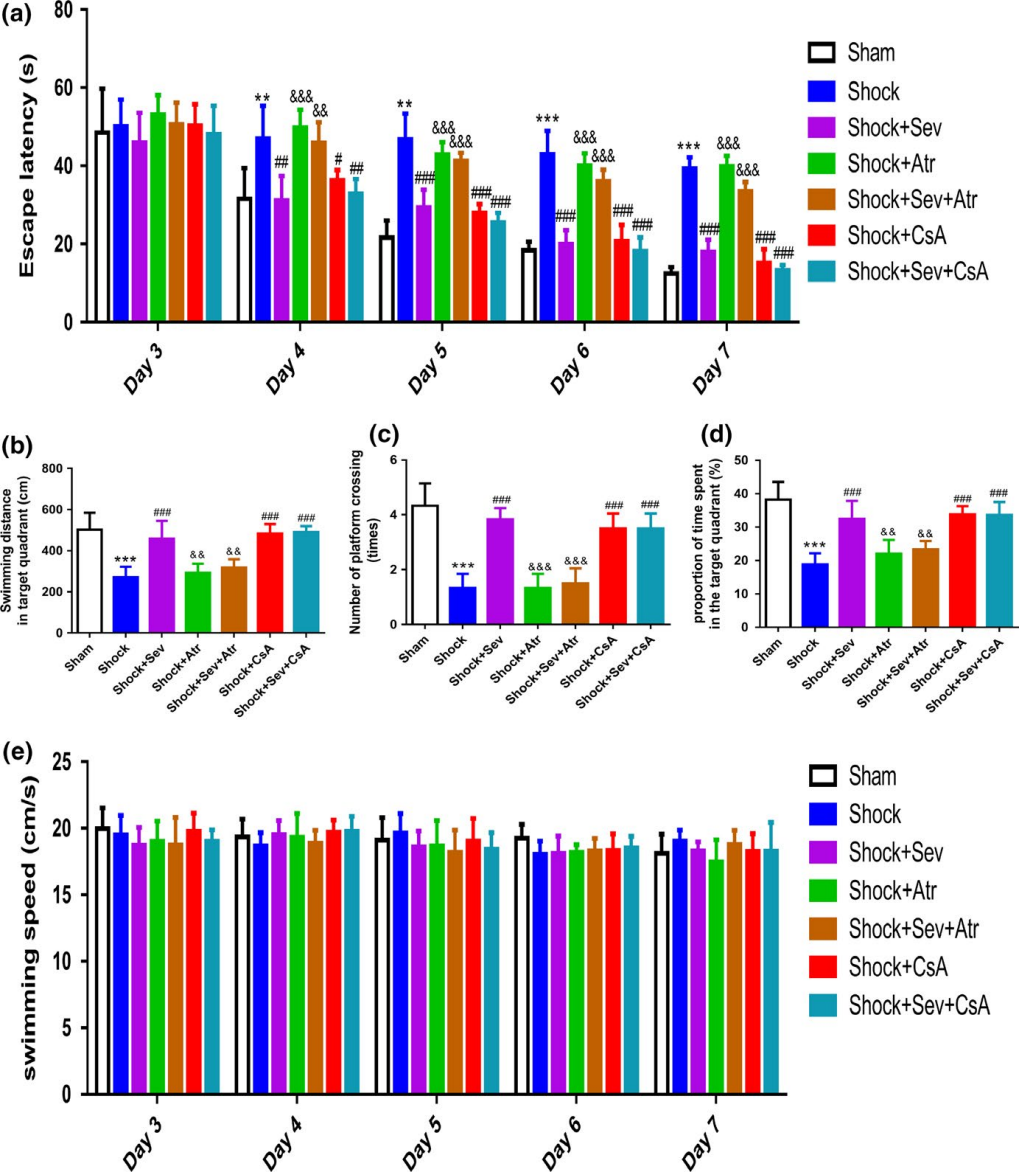
Effects of sevoflurane postconditioning on spatial learning and memory in the Morris water maze. Escape latency (expressed in s; a); swimming distances in target quadrant (expressed in cm; b); numbers of platform crossings (expressed in times; c); proportion of time spent in the target quadrant (expressed in %; d); swimming speed (expressed in cm/s; e). *N* = 6 in each group, data are presented as mean ± standard error of mean, **p < .01 and ***p < .001 versus Sham group, ^#^ *p* < .05, ^##^ *p* < .01, and ^###^ *p* < .001 versus Shock group, ^&&^ *p* < .01 and ^&&&^ *p* < .001 versus Shock+Sev group

To evaluate the effect of sevoflurane postconditioning on spatial learning and memory after hemorrhage shock and resuscitation in rats, we used the Morris water maze (MWM) test 3 days after reperfusion. The escape latency of finding the platform was totally declined in Sham group during the training period. Compared with the Sham group, the escape latency of Shock group was persistently longer throughout the whole training session. From the second learning session till the fifth, compared with Shock group, the Shock+Sev, Shock+CsA, and Shock+Sev+CsA groups always significantly shortened the latency. However, compared with the Shock+Sev group, Shock+Atr and Shock+Sev+Atr groups always significantly prolonged the latency (Figure 3a; *p* < .05, *p* < .01 or *p* < .001). The swimming distance, the number of platform crossings, and the proportion of time spent in the target quadrant in the Shock group were significantly decreased than Sham group. Compared with Shock group, indexes above in Shock+Sev, Shock+CsA, and Shock+Sev+CsA groups were all increased. However, indexes above in Shock+Atr and Shock+Sev+Atr groups were all decreased when compared with Shock+Sev group (Figure 3b,c,d; *p* < .01 or *p* < .001). In addition, no significant differences in swimming speed were found among all groups during the whole experiment (Figure 3e).

### 3.3 Effect of sevoflurane postconditioning on the apoptosis in hippocampus

To investigate the protective effect of sevoflurane postconditioning on the apoptosis in hippocampus, which is associated with the spatial learning and memory, the expression levels of apoptosis proteins (such as Bcl‐2, Bax, and Cyt C) were evaluated by WB, and the activity of caspase‐3 in hippocampus was evaluated by immunohistochemistry analysis.

**Figure 4:**
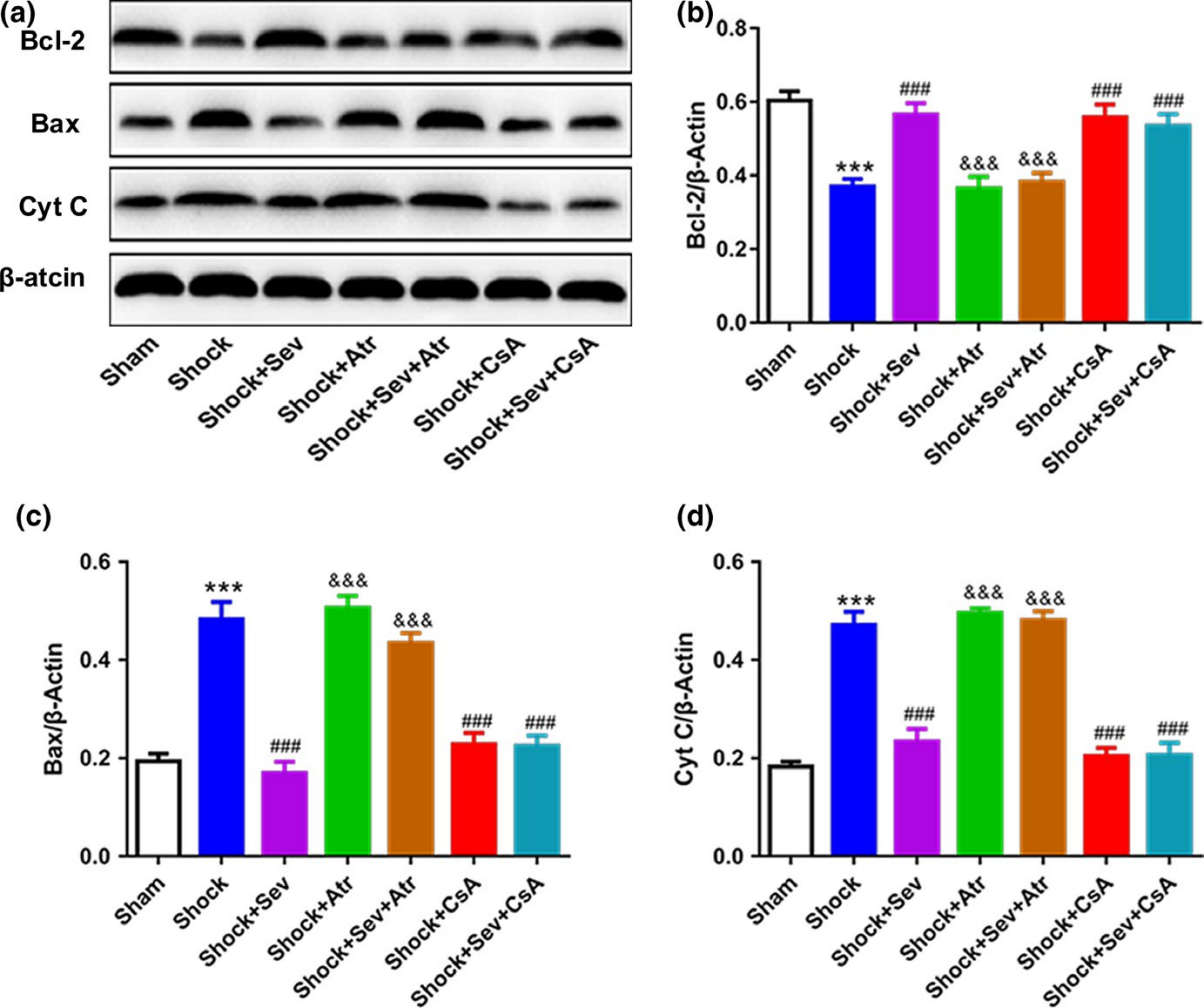
Effect of sevoflurane postconditioning on the expression of protein following hemorrhagic shock and resuscitation. Representative immunoblots of Bcl‐2, Bax, Cyt C, and β‐Actin proteins (a) in hippocampus in all groups. The proteins were determined 24 hr after the intervention. Quantification of the relative expression of Bcl‐2 (b), Bax (c), and Cyt C (d) were detected, and β‐Actin was respectively served as the loading control. *N* = 6 in each group, values are presented as mean ± standard error, ***p < .001 versus Sham group, ^###^ *p* < .001 versus Shock group, and ^&&&^ *p* < .001 versus Shock+Sev group

Western blot results indicated that the level of Bcl‐2 in Shock group was decreased than Sham group. Compared with Shock group, the level of Bcl‐2 was increased in Shock+Sev, Shock+CsA, and Shock+Sev+CsA groups. However, the level of Bcl‐2 in Shock+Atr and Shock+Sev+Atr group was lower than Shock+Sev group (Figure 4a,b; *p* < .001). At the same time, both the results of Bax and Cyt C represented opposite trends with Bcl‐2 among all groups (Figure 4a,c,d; *p* < .001).

**Figure 5:**
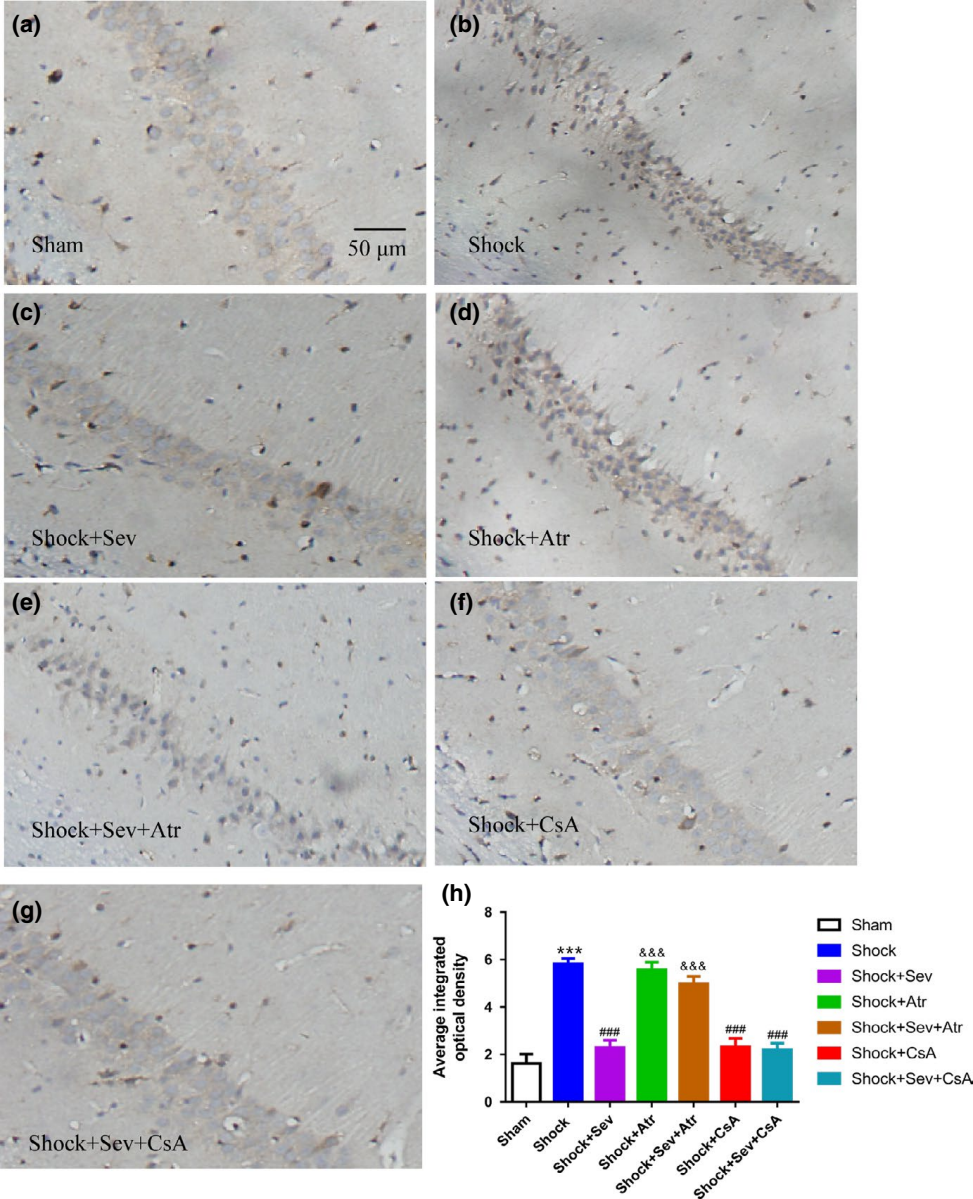
Effect of sevoflurane postconditioning on the staining of caspase‐3 immunoreactive positive cells following hemorrhagic shock and resuscitation. Immunochemical staining of caspase‐3 in the hippocampus CA1 region after the water maze test were shown in the Sham (a), Shock (b), Shock+Sev (c), Shock+Atr (d), Shock+Sev+Atr (e), Shock+CsA (f), and Shock+Sev+CsA (g) groups. Quantitative analysis of caspase‐3 expression in the hippocampal CA1 region was shown in (h). *N* = 6 in each group, data are presented as mean ± standard error, ***p < .001 versus Sham group, ^###^ *p* < .001 versus Shock group, and ^&&&^ *p* < .001 versus Shock+Sev group

Immunochemical staining in the hippocampal CA1 region was light in Sham group but brown in Shock group (Figure 5a,b). The expression of caspase‐3 in Shock+Sev, Shock+CsA, and Shock+Sev+CsA groups was decreased than Shock group (Figure 5b,c,f,g,h;* p* < .001). However, the expression of casapase‐3 was increased in Shock+Atr and Shock+Sev+Atr group compared with Shock+Sev group (Figure 5c,d,e,h; *p* < .001).

### 3.4 Effect of sevoflurane postconditioning on the mPTP opening in the hippocampal CA1 neurons

**Figure 6:**
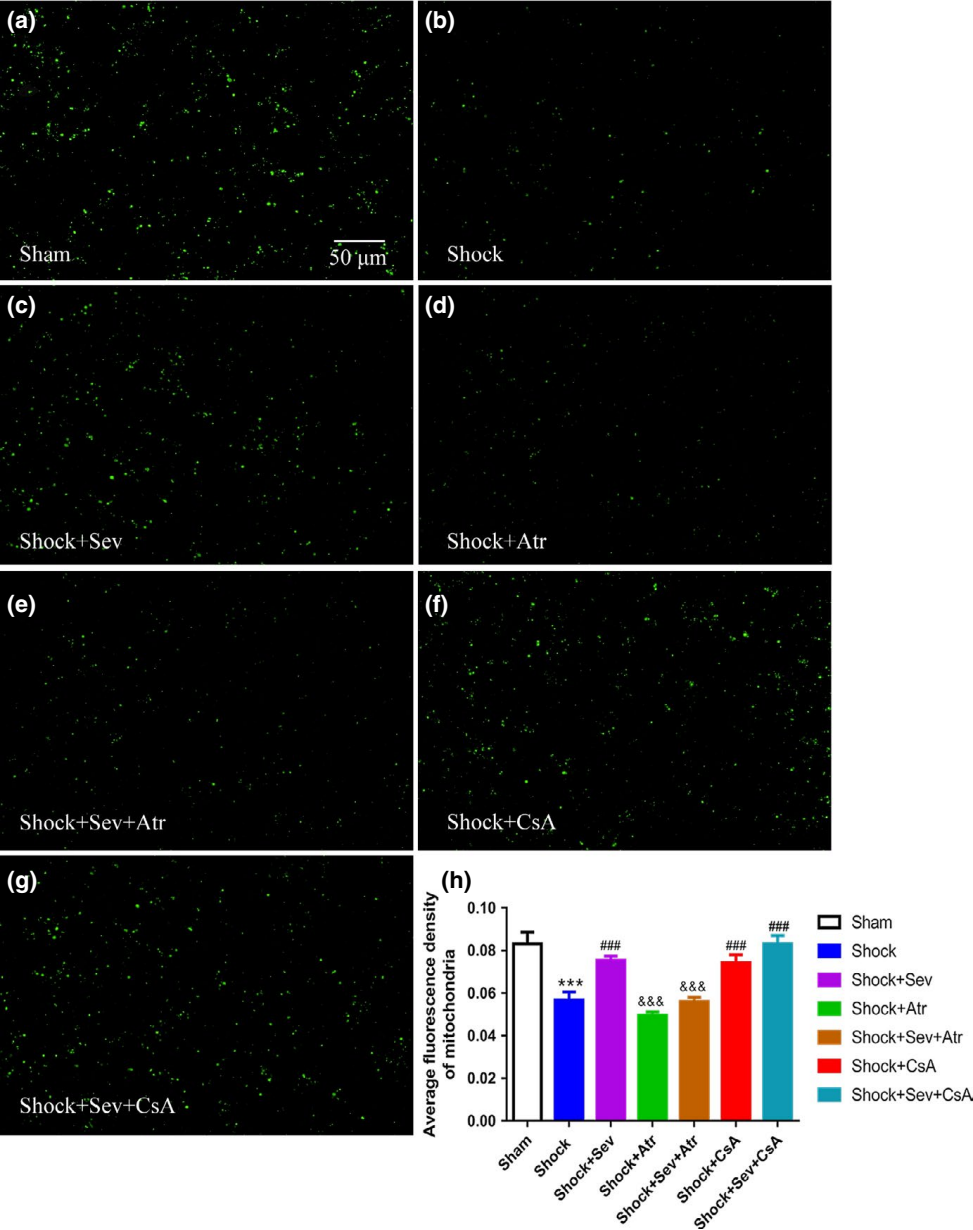
Effect of sevoflurane postconditioning on the opening of mitochondrial permeability transition pore following hemorrhagic shock and resuscitation. The opening of mitochondrial permeability transition pore in isolated mitochondria from hippocampus was measured 24 hr after resuscitation. The changes in mitochondrial permeability transition pore opening were detected with calcein‐AM as a fluorescence indicator by confocal microscopy in the Sham (a), Shock (b), Shock+Sev (c), Shock+Atr (d), Shock+Sev+Atr (e), Shock+CsA (f), and Shock+Sev+CsA (g) groups. Quantitative analysis for the relative changes in calcein fluorescence of the opening of mPTP in the hippocampal CA1 region was shown in (h). *N* = 6 in each group, data are presented as mean ± standard error, ***p < .001 versus Sham group, ^###^ *p* < .001 versus Shock group, and ^&&&^ *p* < .001 versus Shock+Sev group

To further investigate the mechanism of sevoflurane postconditioning eliciting the neuroprotective effect in hemorrhagic shock and resuscitation model, alterations of mPTP opening in the hippocampal CA1 neurons were observed, which were reflected by changes in mitochondrial fluorescence. The average fluorescence intensity of mitochondrial in Shock group was decreased than Sham group (Figure 6a,b,h;* p* < .001), which indicated the degree of mPTP opening in Shock group was higher than Sham group. The average fluorescence intensity of in Shock+Sev, Shock+CsA, and Shock+Sev+CsA groups was all significantly higher compared with Shock group (Figure 6b,c,f,g,h; *p* < .001), which indicated the degrees of mPTP opening in Shock+Sev, Shock+CsA, and Shock+Sev+CsA groups were lower than Shock group. However, the average fluorescence intensity of mitochondrial in Shock+Atr and Shock+Sev+Atr groups was significantly lower than Shock+Sev group (Figure 6c,d,e,h; *p* < .001), which indicated the degrees of mPTP opening in Shock+Atr and Shock+Sev+Atr groups were higher than Shock+Sev group.

### 3.5 Effect of sevoflurane postconditioning on the changes of mitochondrial membrane potential

**Figure 7:**
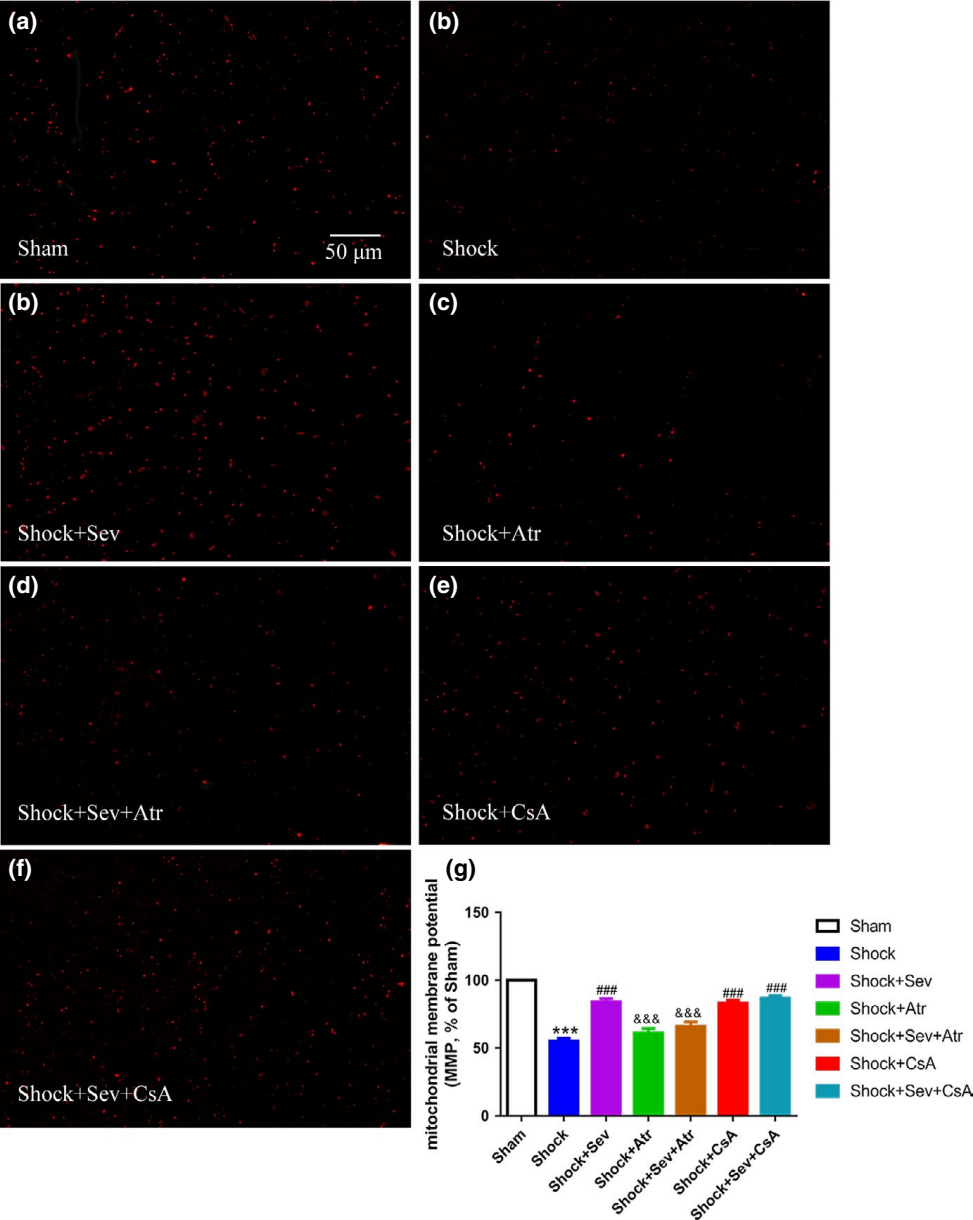
Effect of sevoflurane postconditioning on mitochondrial membrane potential (MMP) following hemorrhagic shock and resuscitation. The mitochondrial membrane potential in isolated mitochondria from hippocampus was measured 24 hr after resuscitation in the Sham (a), Shock (b), Shock+Sev (c), Shock+Atr (d), Shock+Sev+Atr (e), Shock+CsA (f), and Shock+Sev+CsA (g) groups. Summarized data for the relative changes in JC‐1 fluorescence were analyzed in (h). *N* = 6 in each group, data are presented as mean ± standard error, ***p < .001 versus Sham group, ^###^ *p* < .001 versus Shock group, and ^&&&^ *p* < .001 versus Shock+Sev group

To investigate whether alterations of mPTP opening were related to changes of mitochondrial membrane potential (MMP), we used dye of JC‐1 to detect the changes of MMP, which were reflected by changes of the red fluorescence in mitochondria. The MMP level was significantly decreased in Shock group compared with Sham group (Figure 7a,b,h; *p* < .001). The levels of MMP in Shock+Sev, Shock+CsA, and Shock+Sev+CsA groups were significantly higher than Shock group (Figure 7b,c,f,g,h; *p* < .001). However, the levels of MMP in Shock+Atr and Shock+Sev+Atr groups were significantly lower than Shock+Sev group (Figure 7c,d,e,h;* p* < .05).

## 4 DISCUSSION

Using the model of hemorrhagic shock and resuscitation, the results of this study showed that (a) postconditioning with sevoflurane can improve spatial learning and memory ability of hemorrhagic shock and resuscitation rats; (b) sevoflurane postconditioning can inhibit apoptosis by increasing the expression of anti‐apoptotic protein (Bcl‐2), decreasing the expression of pro‐apoptotic proteins (Bax, Cyt C, Caspase‐3), and reducing hippocampal neurons loss; and (c) alterations of mPTP opening and MMP in the hippocampal play an important role in neuroprotective effect of sevoflurane postconditioning.

Acute and severe hemorrhagic shock and resuscitation can cause global body ischemia and reperfusion, leading to myocardial and cerebral dysfunction (Gutierrez, Reines, & Wulf‐Gutierrez, 2004; Wu et al., 2017). In our study, the significant variations of MAP and arterial gas during hemorrhagic shock and resuscitation suggested the severe blood dynamic fluctuation, which is supported by other reports (Fang et al., 2006; Gutierrez et al., 2004). In the resuscitation period, the blood pressure and the results of arterial gas gradually recovery to the level of baselines and all the rats survived. However, sevoflurane postconditioning had no impact on changes of MAP and arterial gas induced by shock (Figure 2), which was consistent with previous studies (Hu, Wang, et al., 2018; Hu et al., 2016; Hu, Zhang, et al., 2018).

Acute severe hemorrhagic shock and fluid resuscitation can lead to neuronal damage (Navarro et al., 2012) or even obvious impairment of spatial learning and memory ability (Hu, Zhang, et al., 2018). We assessed the rats' behavior by Morris water maze test, which is a classic method for testing hippocampus‐related spatial learning and memory ability (Vorhees & Williams, 2006). We found that the escape latency was increased significantly, but the swimming distance, the number of platform crossings, the proportion of time spent in the target quadrant, and the proportion of time spent in the target quadrant were declined significantly in Shock group. Sevoflurane postconditioning or CsA treatment could ameliorate the disability in spatial learning and memory induced by shock, while Atr treatment reversed the protective effect of sevoflurane postconditioning (Figure 3a,b,c,d). The results of Morris water maze were well correlated with the morphology changes of hippocampal CA1 neurons (Figure 5). In addition, this study showed that the swimming speed was similar to each group (Figure 3e), which indicated that the difference of behavioral test was not due to motor impairment.

Neuronal apoptosis is the one of the major neuropathological features during brain ischemic/reperfusion, which was regulated by apoptotic protein family. Bax, Cyt C, and caspase‐3 are pro‐apoptotic proteins while Bcl‐2 is anti‐apoptotic protein. When the cells are subjected to damage, the certain members of the Bcl‐2 family are activated. In the shock model of our study, the expression of Bcl‐2 was reduced, but the expressions of Bax and caspase‐3 were increased in the hippocampus. In contrast, the expression of Bcl‐2 was increased, but the expressions of Bax, Cyt C, and caspase‐3 were decreased by sevoflurane postconditioning or CsA treatment. This result was consistent with previous reports which have confirmed that sevoflurane postconditioning affects the expression of pro‐apoptotic protein and anti‐apoptotic protein (Dong et al., 2014; Liu et al., 2012; Ye et al., 2012). The number of apoptotic neurons was increased in Shock group, but decreased in Shock+Sev, Shock+CsA, and Shock+Sev+CsA groups (Figure 5), which was similar to previous reports (Hu et al., 2016; Lai et al., 2016). However, the effects of reducing neuronal apoptosis in sevoflurane postconditioning or CsA were completely blocked by Atr. Therefore, sevoflurane postconditioning improved spatial learning and memory induced by hemorrhagic shock and resuscitation may be the consequence of the activation of anti‐apoptotic proteins and inhibition of pro‐apoptotic protein, particularly in the hippocampal CA1 region.

Neurons almost completely relied on mitochondria‐supplied ATP to maintain activity and function, so the state of mitochondria is a major important factor that influenced impaired neurons surviving (Kann et al., 2005; Mancuso et al., 2007). mPTP is a nonspecific channel located on the inner membrane of mitochondria, which is comprised of the voltage‐dependent anion channel (VDAC), the adenine nucleotide translocase (ANT), and cyclophilin D (CypD; Akopova, 2008). Many apoptotic stimuli can cause the opening of mPTP, which is an important factor to decide the extent of cell damage (Korde et al., 2007; Wang et al., 2015) and regarded as the initial reason of the mitochondrial dysfunction (Lipton, 1999). Under physiological conditions, mPTP remains closed or rarely opened, but some factors can increase the opening of the mPTP during the hemorrhagic shock and resuscitation, which includes a rise of tissue Ca^2+^ levels, a decrease of ATP levels, a burst of oxidant stress, and further influx of calcium (Hausenloy, 2003). Sevoflurane postconditioning against cerebral ischemia–reperfusion injury was proved to involve inhibition of mPTP opening (Wang et al., 2015). In addition, inhibition of mPTP opening in neuron has already become a neuroprotection strategy (Kristal et al., 2004). Thus, neuroprotection of sevoflurane postconditioning against hemorrhagic shock and resuscitation may also act on mPTP opening.

The level of the mitochondrial membrane potential directly reflects the mitochondrial function and the degree of the opening of mPTP (Huang et al., 2012). In the apoptosis induced by different factors, the decrease of MMP level occurred before the change of cell morphology. Once the mitochondrial membrane potential disappears, the cells will enter an irreversible apoptotic process (Bonnet et al., 2007). In our results, the opening of mPTP was significantly higher and the MMP level was decreased in Shock group compared with Sham group, indicating that alterations of mPTP opening were related to changes of MMP. Compared with Shock group, MMP increased and degree of mPTP decreased in sevoflurane or CsA treatment groups, whereas the effect of sevoflurane was abrogated by Atr treatment. In line with the study of sevoflurane protecting against focal cerebral ischemia and reperfusion injury (Wang et al., 2015), we found that the effect of sevoflurane to reduce neuronal apoptosis is through inhibiting mPTP opening and increasing MMP.

Cyclosporin A is a well‐recognized mPTP inhibitor that mimics the protective effects of sevoflurane postconditioning by preventing CypD to ANT (Lai et al., 2016; Ye et al., 2012). In previous studies, the neuroprotective effect of CsA on the cerebral ischemia has also been confirmed in the rat MCAO model (Leger et al., 2011). In contrast, the neuroprotection of sevoflurane was completely blocked by Atr, which is an ANT ligand (Roux et al., 1996). Studies have shown that Atr can eliminate the inhibiting effect of CsA on mPTP opening via preventing the conformational change (Basso, Petronilli, Forte, & Bernardi, 2008). In this study, changes of mPTP opening and MMP between Shock+Sev and Shock+Sev+Atr groups proved that Atr eliminated the neuroprotective effect of postconditioning with sevoflurane by blocking mPTP opening. However, no significant difference between Shock and Shock+Atr groups suggests that Atr alone had no effect on diminishing shock‐induced ischemia and reperfusion injury, which is consistent with previous study (Wang et al., 2015). The results in Shock and Shock+CsA groups showed that CsA could inhibit the mPTP opening. In addition, no significant differences among Shock+Sev, Shock+CsA, and Shock+Sev+CsA groups showed that CsA played the similar protective role as sevoflurane postconditioning. Our results suggest that sevoflurane postconditioning‐induced neuroprotection is mediated by the mPTP.

It is well known that Atr has nephrotoxicity and hepatotoxicity (Daniele et al., 2005). Studies have shown that Atr induces renal tubular necrosis at high doses and apoptosis at lower doses (Obatomi, Blackburn, & Bach, 2001). The toxicity of Atr is unavoidable, but this may be dose‐dependent. CsA also has been shown to induce serious renal and hepatic side effects, which is reported that subcutaneous administration of 25 mg/kg CsA for 5 weeks can induce nephropathy in rats (Patel & Thaker, 2015). However, the dose of CsA (10 mg/kg) in our study is less than the dose (25 mg/kg) which produced nephrotoxicity. Therefore, the selected doses of atractyloside (Atr, 5 mg/kg) and cyclosporine A (CsA, 10 mg/kg) are supported by previous references (Ma et al., 2018; Wang et al., 2015; Ye et al., 2012), which have been proved to be the effective doses in study of mPTP.

Sevoflurane postconditioning is a better clinical option than other pretreatment methods because it does not need to be executed before ischemia, but can be administered during reperfusion. Nevertheless, there are some limitations to this study. Firstly, arterial blood gas and MAP were not measured all the time. Secondly, 2, 3, 5‐triphenyltetrazolium chloride (TTC) and TUNEL staining were not operated, although we have observed the apoptosis of hippocampal cells in other ways. Thirdly, we established hemorrhagic shock model by withdrawing blood via carotid artery at a constant speed but the applicability of the patients remains to be confirmed.

In conclusion, sevoflurane postconditioning improved the ability of spatial learning and memory after hemorrhage shock and resuscitation in rats. The neuroprotection of sevoflurane postconditioning is possibly mediated by blocking the mitochondrial permeability transition opening, increasing mitochondrial membrane potential, and reducing neuronal apoptosis in the hippocampus of hemorrhagic shock and resuscitation rats.

## CONFLICT OF INTEREST

The authors declare no conflict of interests.

## AUTHOR CONTRIBUTIONS

Data collection, data analysis, and manuscript writing: ZL, HL; experiment implementation: WJX, ZHC; experimental design and prudential comments on the whole process of this work: ZY, HXW. All authors have read and approved the final version of this paper.

## Data Availability

The data that support the findings of this study are available from the corresponding author upon reasonable request.
